# Iron deficiency affects oxygen transport and activates HIF1 signaling pathway to regulate phenotypic transformation of VSMC in aortic dissection

**DOI:** 10.1186/s10020-024-00859-y

**Published:** 2024-06-17

**Authors:** Yuanyang Chen, Xu Li, Zhiwei Wang, Shun Yuan, Xiaoyan Shen, Xiaoping Xie, Kai Xing, Qingyi Zhu

**Affiliations:** 1 Department of Cardiothoracic Surgery, Renmin Hospital of Wuhan University, Wuhan, Hubei People’s Republic of China; 2 Cardiovascular Surgery Laboratory, Renmin Hospital of Wuhan University, No. 9 Zhangzhidong Road, Wuhan, 430000 Hubei People’s Republic of China

**Keywords:** Aortic dissection, Aortic media degeneration, Iron deficiency, HIF1, Phenotypic transformation

## Abstract

**Background:**

Aortic dissection (AD) is a macrovascular disease which is pathologically characterized by aortic media degeneration.This experiment aims to explore how iron deficiency (ID) affects the function of vascular smooth muscle cell (VSMC) and participates in the occurrence and development of AD by regulating gene expression.

**Methods:**

The relationship between iron and AD was proved by Western-blot (WB) and immunostaining experiments in human and animals. Transcriptomic sequencing explored the transcription factors that were altered downstream. WB, flow cytometry and immunofluorescence were used to demonstrate whether ID affected HIF1 expression through oxygen transport. HIF1 signaling pathway and phenotypic transformation indexes were detected in cell experiments. The use of the specific HIF1 inhibitor PX478 further demonstrated that ID worked by regulating HIF1.

**Results:**

The survival period of ID mice was significantly shortened and the pathological staining results were the worst. Transcriptomic sequencing indicated that HIF1 was closely related to ID and the experimental results indicated that ID might regulate HIF1 expression by affecting oxygen balance. HIF1 activation regulates the phenotypic transformation of VSMC and participates in the occurrence and development of AD in vivo and in vitro.PX478, the inhibition of HIF1, can improve ID-induced AD exacerbation.

## Highlights

1. This study demonstrated for the first time that ID can alter HIF1 transcription through oxygen transport and participate in the occurrence of AD.
2. The promoting effect of ID on phenotypic transformation of VSMC may be through HIF1 signaling pathway.
3. Specific inhibition of HIF1 can effectively alleviate the exacerbation of AD disease caused by ID and HIF1 may be an important target for drug therapy of AD.

## Introduction

Aortic dissection (AD) is a life-threatening condition caused by a tear in the intimal layer of the aorta or bleeding within the aortic wall, resulting in the separation (dissection) of the layers of the aortic wall (Akutsu 2019). Aortic dissection is most common in individuals aged 65–75 years, with an incidence rate of 35 cases per 100,000 people per year in this population (Evangelista et al. 2018). Aortic media degeneration (AMD), characterized by cytoskeleton destruction and dysfunction of aortic smooth muscle cells (ASMCs), as well as phenotypic switching and apoptosis, is considered to be the specific condition that promotes AD development (Shen et al. 2020). How to regulate the occurrence and development of ADby influencing the structure and function of smooth muscle cells has always been a hot topic in cardiovascular surgery research and finding suitable targets to delay the occurrence of AD is what we pursue.

Trace element iron plays crucial roles in various biochemical processes within organisms, such as hemoglobin synthesis and peroxidase activation (Shi and Wang 2020). Iron deficiency (ID) can activate multiple signal transduction pathways and regulate oxidative stress, phenotypic remodeling and inflammatory response of vascular smooth muscle cells (VSMC), thereby affecting AMD development (Li et al. 2021).There is substantial evidence supporting the central involvement of hypoxia-inducible factors (HIFs) in the iron-deficient environment (Camaschella 2019); however, there is currently no clear evidence demonstrating how HIFs are involved in the pathological process underlying AD occurrence and development. Hypoxia-inducible factor 1 (HIF1) is the central actor of an ancient, highly conserved pathway that responds to low-oxygen conditions. This transcription factor is composed of two subunits: constitutively expressed HIF1β and oxygen-sensitive HIF1α.In normoxic conditions, the HIF prolyl hydroxylases (PHDs) and asparaginyl hydroxylase (Factor Inhibiting HIF or FIH, respectively) modify residues on HIF1α that target the protein for degradation and prevent its transcriptional activity. During hypoxia, these posttranslational modifications are limited, allowing HIF1α to enter the nucleus, dimerize with HIF1β, and bind to genomic hypoxia response elements to promote transcription (Knutson et al. 2021).When it combines with target genes, the body produces a series of reactions through transcription and post-transcriptional regulation, some of which, despite the nature of adaptive compensation, often cause pathological damage to the body, such as hypoxic pulmonary hypertension (HPH) and accelerated tumor growth (Cyran and Zhitkovich 2022; Chen et al. 2019). Therefore, overactivation of HIF1 signal may promote the occurrence of AD by affecting vascular function.

The purpose of this study is to explore the direct correlation between ID and HIF1, and how ID affects the phenotypic transformation of VSMC through HIF1 to participate in the development of AD, so as to provide more feasible research ideas for the treatment of AD.

## Materials and methods

### Human tissues

The experimental ethics were reviewed and approved by the Clinical Research Ethics Committee of Renmin Hospital of Wuhan University(China). All human specimens were used with the informed written consent of all patients and donors (WDRY2020-K230). Six aortic specimens were obtained from patients with type A aortic dissection who underwent aortic replacement surgery between April 2021 and August 2023 and who did not exhibit any phenotypic characteristics of known genetic disorders, such as Marfan syndrome and loeysdietz syndrome. In addition, 6 samples of normal aorta were obtained from brain-dead patients or heart transplant patients who were registered as heart donors. Human aorta samples are mainly used for immunostaining and western-blot.

### Animals and cells

Male C57 mice (average weight: 12 g; age: 3 weeks) were purchased from Mouse Treasure Corporation (China). Mice were randomly assigned to each group (6 mice per group) and mice were fed a special diet containing 0.25% β-Aminopropionitrile (BAPN) (Yuanye company, S44439, China) to construct an AD model (Pan et al. 2022). Feed for 4 weeks to build AD model and when some mice died during the course, the samples were fixed in time. BAPN feed and iron-deficient feed were purchased from Beijing Huafukang Company. All animal experiments were approved by the Ethics Committee of Renmin Hospital of Wuhan University (WDRM20201107).The mouse aorta smooth muscle cell line of MOVAS (Enzyme-link Biotechnology, ML096614, Shanghai) was used in cell experiments (Xiao et al. 2023). The cells were cultured in a 37 degree incubator with 5% CO_2_. Angiotensin-II (Ang-II) reatment of MOVAS cells can induce their transformation from contractile to synthetic, simulating the process of AD. Deferoxamine (DFO) is an iron chelating agent that reduces the deposition of free iron ions in cells and simulates iron-deficient environments.

### Transcriptomic sequencing

Whole transcriptomic sequencing was performed on freshly obtained human aorta specimens (3 independent samples per group), and the sequencing technology was provided by Sangon Biotech (Shanghai). After analyzing the sequencing results, the treated MOVAS cells were sequenced again in order to exclude the large differences in the human tissue itself (3 independent samples per group). The two sequencing results were basically consistent, which verified the reliability of the experiment.Ten thousand sequences were randomly selected from Clean data and compared with NCBI NT database for blastn comparison. Comparison results with evalue <  = 1e−10, similarity > 90% and coverage > 80% were used to calculate their species distribution.

### Flow cytometry

To detect the expression changes of ROS, iron and HIF1s (CST,#36,169,USA) in vivo and in vitro experiments, flow cytometry was performed on mouse blood and movas cell lines after modeling.

Peripheral blood was collected from the eye socket and red blood cells were removed with red blood cell lysate to eliminate the influence of blood color on fluorescence intensity.HIF1 antibody and iron ion fluorescent probe (Solarbio,#121,714,China) were used for 37 °C dark staining 30 min.

The evaluation of ROS changes in movas cells involved the use of flow cytometry to detect dihydroethidine (DHE) (Beyotime, #S0063, China) probe for measuring ROS concentrations. The fluorescence intensity (FI) emitted by DHE was quantified in the control group, and initially, DHE (blank) was not added during analysis to ensure undisturbed fluorescence emission.

### Immunohistochemistry/immunofluorescence

To assess the expression and subcellular localization of relevant proteins in aortic smooth muscle cells, immunostaining was performed on aortic sections. After the sections were hydrated with xylene and gradient alcohol, they were repaired by microwave heating in sodium citrate buffer (Servicebio,#G1201,Wuhan). The sections were sealed with goat serum and incubated overnight at 4 ℃ for primary antibody. After incubation of the corresponding goat secondary antibody the next day, DAB (Servicebio, #G1212, Wuhan) was used for positive signal color development DAPI (Servicebio, #G1012, Wuhan) was used for immunofluorescence staining). A standing microscope was used to take pictures under different fields of view (Image magnification is 10× and 40×, the confocal shooting multiple is 200×) and the acquired pictures were analyzed by Image J software.

### Western blotting (WB)

Tissues and cells were washed with a PBS and lysed in RIPA buffer (Beyotime, #P0013K, China) containing cocktail (Servicebio, #G2006-250UL, China) and phenylmethylsulfonyl fluoride (Servicebio, #G2008-1ML, China) on ice. Tissue fragments and cell debris were extracted by centrifugation(10,000*g*, 4 °C, 12 min) after ultrasonication, and the protein concentration of the supernatant was determined by a BCA assay (Beyotime, #P0010, China). Equal amounts of proteins (MOVAS, 20 μg; human tissue, 200 μg; and mouse aortic tissue, 120 μg) were resolved by 8%–10% SDS PAGE and transferred onto polyvinylidene difluoride (PVDF) membranes (Merck Millipore, # ISEQ00010, USA). After being blocked with 5% skimmed milk in PBST for 1.5 h, the membranes were incubated overnight with anti-HIF1 antibodies (1: 600,CST,#36,169, USA), anti-VEGF antibodies (1:500, ThermoFisher, MA5-13,182, USA), anti-iNOS antibodies (1: 600,CST,#2982, USA), anti-eNOS antibodies (1: 600,CST,#5880, USA) anti-α-SMA antibodies (1: 800, Servicebio, #GB111364, China), anti-SM-22α antibodies (1: 1000; Beyotime, #AF5318, China), anti-OPN antibodies (1: 1000; Proteintech, #22,952–1-AP, USA), anti-MMP2 antibodies (1: 1000; Servicebio, #GB11130, China), anti-MMP9 antibodies (1: 1000; Beyotime, #AF5234, China), and anti-β-actin (1: 1000; Proteintech, #66,009–1-Ig, USA) primary antibodies at 4 °C. The membranes were washed and incubated with IRDye-800CW-conjugated goat anti-mouse IgG (1: 20 000; Li-Cor, #926–32,210, USA) or goat anti-rabbit IgG (1: 20 000; Li-cor, #925–32,211, USA) and HRP-conjugated goat anti-mouse IgG (1: 20 000; Li-cor, #926–80,010, USA) or goat anti-rabbit IgG (1: 20 000; Li-Cor, #926–80,011, USA) secondary antibodies. The membranes were scanned using an Odyssey (Li-Cor Biosciences, USA) and chemiluminescence apparatus (BIO-RAD, USA), and the grayscale value of each band was qualified using paired software. At least 3 independent experiments were performed.

### Masson and Elastic van Gieson

Aortic tissue was fixed, dehydrated and embedded in paraffin, and sectioned as described above. As for Masson staining, sections were stained with hematoxylin (Servicebio, #G1004, Wuhan) solution for 10 min, differentiated with 1% hydrochloric acid ethanol, and then stained with ponceau acid fuchsin solution, phosphomolybdic acid solution and aniline blue solution (Servicebio, #G1032, Wuhan) successively. Subsequently, sections were dehydrated with gradient ethanol, made transparent with xylene, and mounted with neutral resin. Images were captured by an optical microscope. The red part represents elastic fibers and the blue part represents collagen.

As for Elastic van Gieson (EVG) staining, alcohol hematoxylin, ferric chloride, and iodine solution were mixed with EVG dye solution according to a certain proportion. The sections were immersed in the EVG dye solution for 30 min and then rinsed under running water. Iron trichloride differentiation liquid differentiated, to the elastic fibers appeared dark purple against an almost colorless background. Saturated picric acid was mixed with ponceau S (Servicebio, #G2011, Wuhan)stain to form the EVG dye solution. Sections were stained for 1–3 min, then rapidly washed, dehydrated, and mounted.

### Statistical analysis

All statistical analyses were performed using GraphPad Prism 6.0 (GraphPad Software, USA), and the results are presented as the mean ± standard deviation (SD). Mouse survival was compared using the logrank test. For ex vivo experiments, the analysis of variance (one-way ANOVA) followed by Dunnett’s test was used to compare differences among multiple groups. Other measurements were performed using unpaired Student’s t-test. P < 0.05 were considered statistically significant.

## Results

### Iron deficiency increases the incidence of aortic dissection and aggravates the degree of aortic lesions

**Fig. 1:**
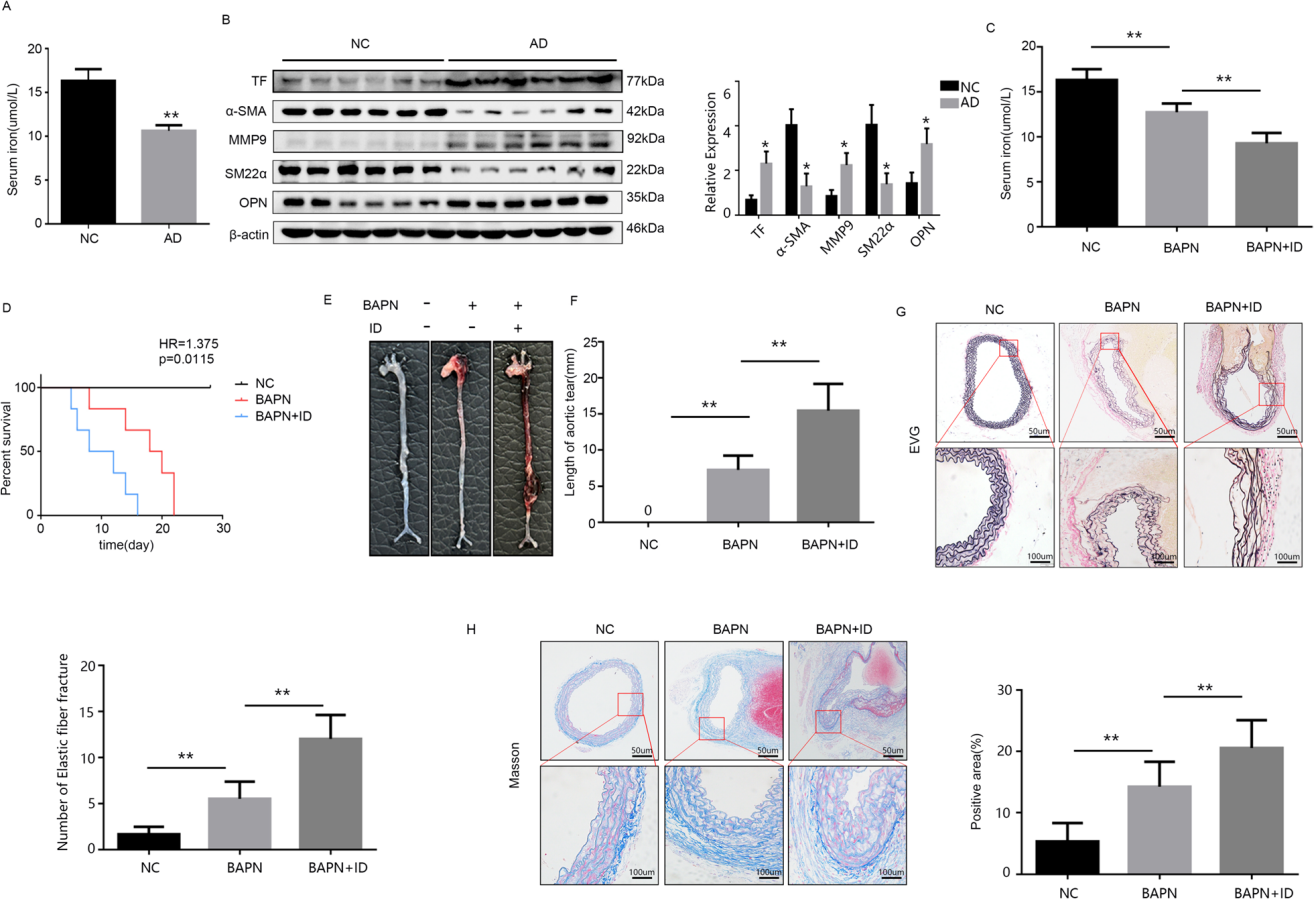
ID promotes the development of AD and increases the extent of the lesions. **A** Serum iron content of patients (n = 6). **B** Western-blotting results and statistical analysis of relevant indicators in human. **C** Detection of serum iron in modeling mice (n = 6). **D** Survival curve of mice. **E** Appearance of mouse aorta. **F** Lesion involvement length. **G** and **H** EVG and Masson staining results and statistics.All data were presented as mean ± SD. Survival curve was compared using the logrank test (HR = hazard ratio). ns means that thereis no statistical significance, **p < 0.01, *p < 0.05

Free iron ions were detected in the serum of 12 patients, and a significant reduction in serum iron levels was observed among individuals with AD, providing evidence for the presence of iron deficiency in AD patients (Fig. 1A). The human aortic protein was extracted for Western blot analysis, which revealed an up-regulation of TF expression in response to iron deficiency and significant alterations in indices associated with AD (Fig. 1B). After establishing an animal model of iron deficiency, we quantified the levels of serum iron ions in mice and observed a significant reduction associated with an iron-deficient diet, thereby validating our modeling outcomes (Fig. 1C). Survival curves (Fig. 1D) demonstrated that an iron-deficient diet exacerbated BAPN-induced aortic dissection occurrence, making it more challenging for mice to survive. The ruptured aorta was imaged for comparative analysis and the extent of aortic involvement in aortic dissection was quantified for all mice (Fig. 1E and F). The findings revealed that iron deficiency exacerbated the extent of aortic dissection lesions and amplified the area of aortic rupture. The aorta of the mouse was subjected to EVG and Masson staining for the analysis of pathological alterations (Fig. 1G and H). The findings revealed that iron deficiency compromised the structural integrity by augmenting smooth muscle fiber breakdown while diminishing their content.

### Iron deficiency leads to oxidative stress and increases HIF1 expression

**Fig. 2:**
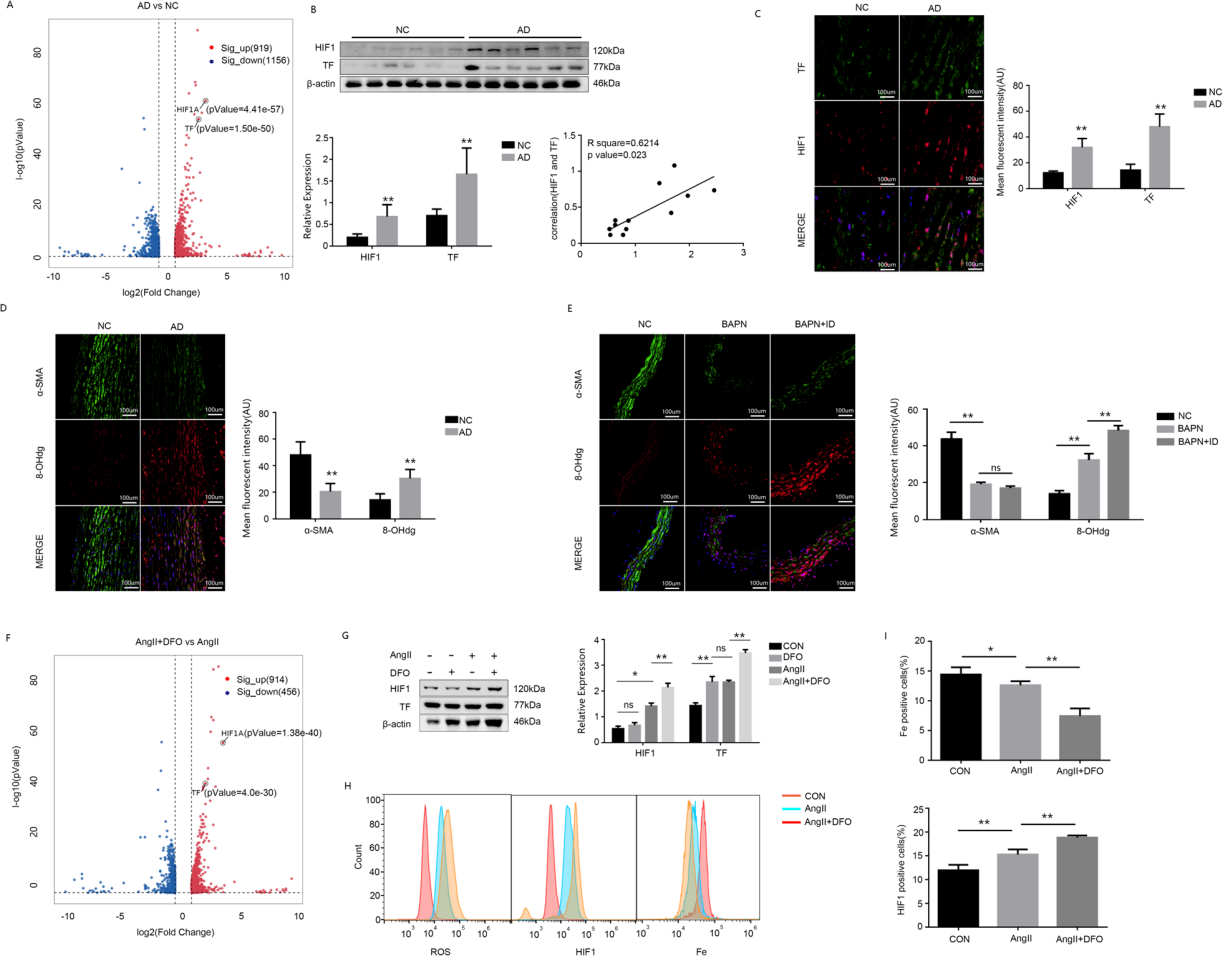
ID promotes the expression of HIF1 through inducing oxidative stress. **A** Human transcriptomic sequencing results. **B** WB detection and analysis of HIF1 and TF in human. **C** Immunofluorescence of HIF1 and TF in human aortic. **D** Immunofluorescence of α-SMA and 8-OHdg in human aortic. **E** Immunofluorescence of α-SMA and 8-OHdg in mouse aortic. **F** MOVAS transcriptomic sequencing results. **G** WB detection and analysis of HIF1 and TF in MOVAS. **H** The contents of ROS, HIF1 and iron were determined by cytometry. **I** Quantification of iron ion levels and enumeration of HIF1-positive cells. Data are representative of three independent experiments and presented as mean ± SD. ns means that thereis no statistical significance, **p < 0.01

Initially, transcriptomic sequencing was performed on human specimens to investigate potential downstream alterations (Fig. 2A). The sequencing results revealed a significant increase in the expression of HIF1 and TF in the AD group, indicating a strong correlation between iron deficiency and HIF1 expression. Subsequently, protein extraction from the human aorta was performed to validate the sequencing results at the protein level, accompanied by an investigation into the correlation between HIF1 and TF (Fig. 2B). The Western blot experiments revealed a significant up-regulation of TF and HIF1 expression in the aorta of the AD group, demonstrating a positive correlation. Immunofluorescence staining of human aorta sections confirmed the findings obtained from WB experiments, thereby further validating the elevated expressions of TF and HIF1 observed in the sequencing results (Fig. 2C). To validate the induction of oxidative stress by iron deficiency, we assessed alterations in 8-OHdg levels (a reliable marker for oxidative stress) within aortic specimens obtained from both human subjects and animal models (Fig. 2D and E). The immunofluorescence results revealed a significant increase in 8-OHdg expression and elevated levels of oxidative stress induced by iron deficiency. To minimize inter-individual tissue variations in human specimens during experimentation, transcriptome sequencing was conducted on movas cells subjected to diverse experimental conditions (AngII + DFO vs AngII). In line with our findings derived from human sequencing data, both HIF1 and TF expressions exhibited a significant up-regulation in the context of iron deficiency (Fig. 2F). The protein extraction from cells for Western blot detection revealed a positive correlation between TF and HIF1 expression (Fig. 2G), suggesting that iron deficiency may contribute to the up-regulation of HIF-1 expression induced by oxidative stress. The flow cytometry analysis conducted on iron-deficient cells revealed a significant increase in intracellular levels of reactive oxygen species (ROS), accompanied by an up-regulation of HIF1 expression (Fig. 2H and I).

### HIF1 signal activation leads to phenotypic transformation of smooth muscle cells in vivo

**Fig. 3:**
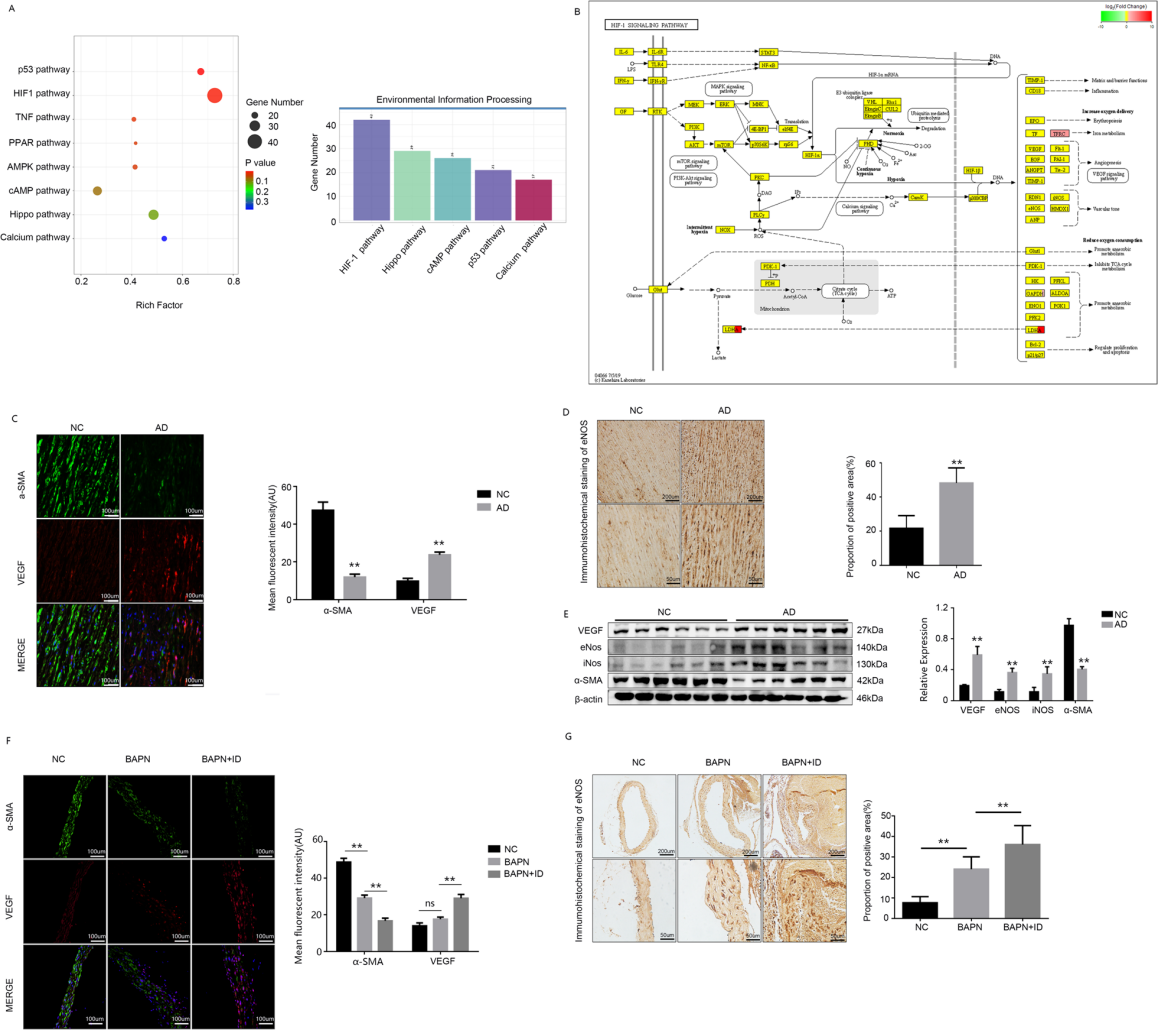
HIF1 signaling activation promotes phenotypic transformation of VSMC in vivo. **A** KEGG analysis of human aorta. **B** HIF1 signaling pathway. **C** Immunofluorescence of α-SMA and VEGF in human aorta. **D** Immunohistochemistry of eNOS in human aorta. **E** WB detection and analysis of HIF1 pathway protein in human. **F** Immunofluorescence of α-SMA and VEGF in mouse aorta. **G** Immunohistochemistry of eNOS in mouse aorta. Data are representative of three independent experiments and presented as mean ± SD. ns means that thereis no statistical significance, **p < 0.01

According to the results of KEGG analysis (Fig. 3A), there is a significant alteration observed in the HIF1 signaling pathway in AD patients, which may be intricately associated with the pathogenesis and progression of this disorder. According to the sequencing results, there were significant alterations observed in the downstream proteins of the HIF1 signaling pathway (Fig. 3B). The expression of HIF1 in the human aorta was preliminarily correlated with α-SMA, an established marker for VSMC transformation, as determined by immunofluorescence and Immunohistochemistry detection (Fig. 3C and D). The human aortic protein was detected using Western blot analysis, revealing a significant up-regulation in downstream protein expression within the HIF1 signaling pathway (Fig. 3E). Immunohistochemistry and immunofluorescence staining were performed in animal aorta sections (Fig. 3F and G), the staining results showed that iron deficiency could activate HIF1 signaling pathway, promote the transformation of aortic smooth muscle cells into synthetic and accelerate the progression of AD.

### The activation of HIF1 leads to the phenotypic transformation of VSMCs through VEGF in vitro

**Fig. 4:**
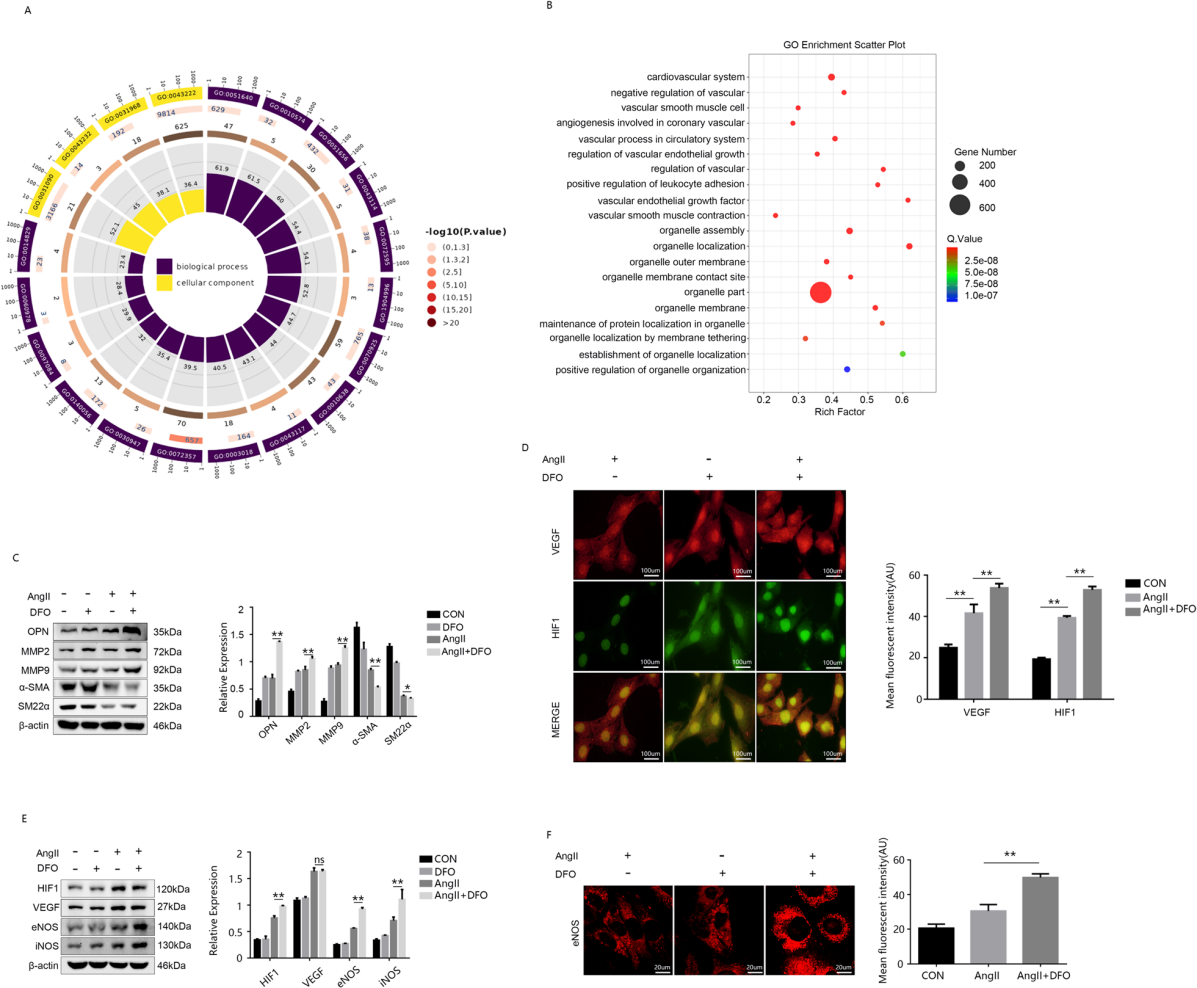
HIF1 activation significantly altered phenotypic transformation of VSMC in vitro. **A** GO analysis of MOVAS. **B** KOG analysis of MOVAS. **C** WB detection and analysis of phenotypic transformation-related proteins.**D** Immunofluorescence of HIF1 and VEGF in MOVAS. **E** WB detection and analysis of HIF1 signaling. **F** MOVAS under confocal microscope. Data are representative of three independent experiments and presented as mean ± SD. ns means that thereis no statistical significance, **p < 0.01

According to the Gene Ontology (GO) analysis conducted after cell sequencing, iron deficiency was found to primarily induce alterations in cellular processes and components (Fig. 4A). Specifically, the GO analysis indicated significant alterations in vascular function and structure, as well as modifications and remodeling of cellular intima organs (Fig. 4B). To validate these sequencing results, WB detection was performed on extracted cells which confirmed that HIF activated VEGF expression promotes phenotypic transformation of smooth muscle cells in vitro (Fig. 4C). Immunofluorescence co-localization was employed to demonstrate the interaction between HIF1 and VEGF (Fig. 4D). WB experiments indicated a significant increase in expression of the HIF1 signaling pathway after ID treatment (Fig. 4E). Following ID + AngII treatment, cellular morphology changed from elongated shuttle-like shape to oval shape while exhibiting a significant alteration in HIF1 downstream signaling pathway (eNOS) as well (Fig. 4F).

### Inhibition of HIF1 can attenuate the phenotypic transformation of VSMC and ameliorate the occurrence and progression of AD

**Fig. 5:**
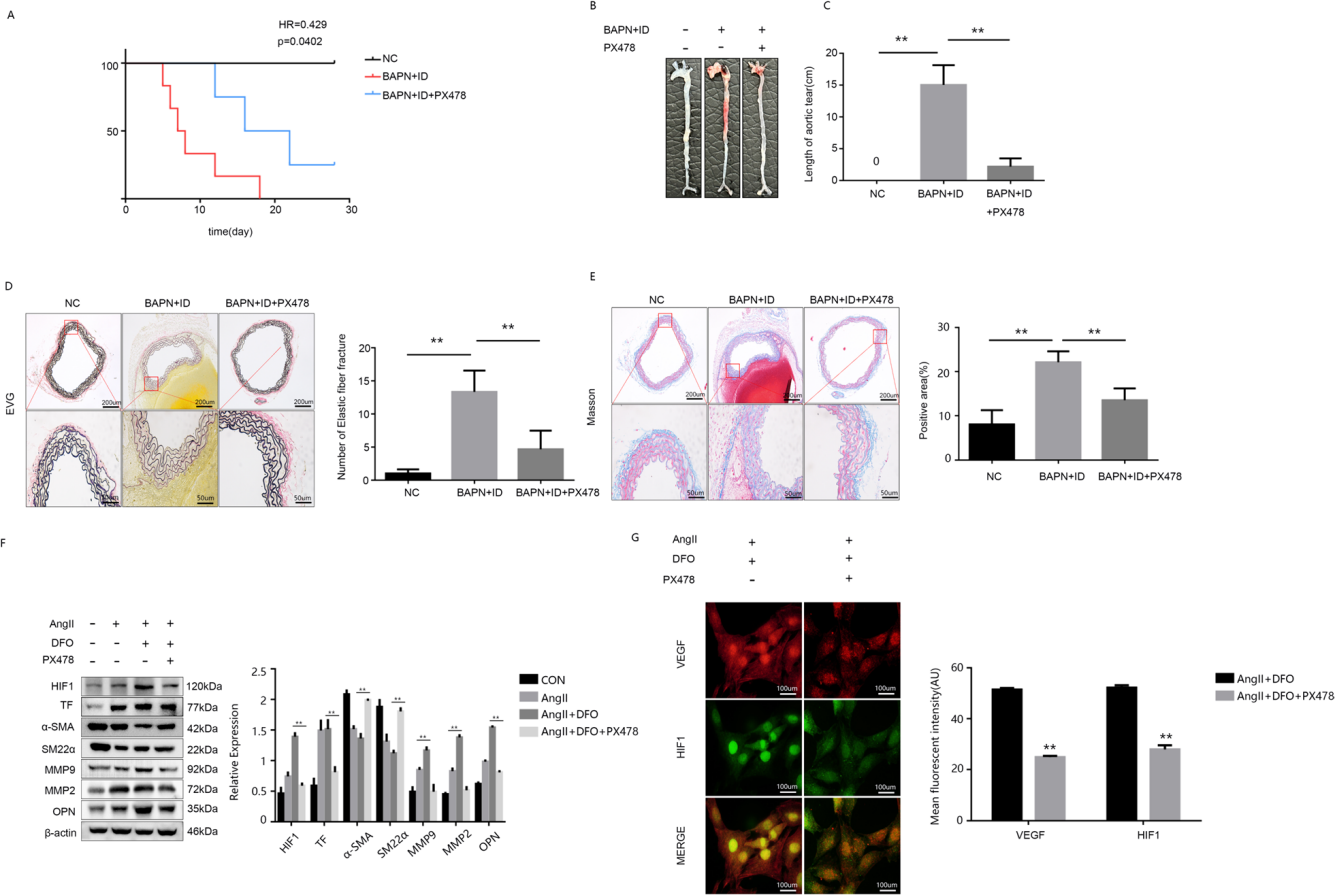
Inhibition of HIF1 can attenuate the phenotypic transformation of VSMC and ameliorate the occurrence and progression of AD. **A** Survival curve of mice. **B** Appearance of mouse aorta. **C** Lesion involvement length. **D** and **E** EVG and Masson staining results and statistics. **F** WB detection and analysis of phenotypic transformation and HIF1 signaling pathway related proteins. **G** Immunofluorescence of HIF1 and VEGF in MOVAS. Data are representative of three independent experiments and presented as mean ± SD. ns means that thereis no statistical significance, **p < 0.01

The survival analysis revealed that PX478, a specific inhibitor of HIF1, significantly extended the survival period in mice with ID + AngII-induced AD (Fig. 5A). External examination revealed that PX478 reduced the incidence of aortic dissection and mitigated the extent of dissection rupture (Fig. 5B and C). EVG and Masson staining results also indicated that PX478 improved elastic fiber integrity and decreased elastic collagen deposition in mouse aortas (Fig. 5D and E).The WB analysis in vitro experiments demonstrated the suppressive effect of PX478 on the expression levels of phenotypic transformation markers in smooth muscle cells, while showing no significant impact on iron metabolism (Fig. 5F). Moreover, the inhibition of HIF1 was validated by cell fluorescence staining, which demonstrated a significant reduction in VEGF expression. This finding further strengthens the potential of HIF1 inhibitors to enhance smooth muscle cell phenotypic transformation (Fig. 5G).

## Discussion

**Fig. 6:**
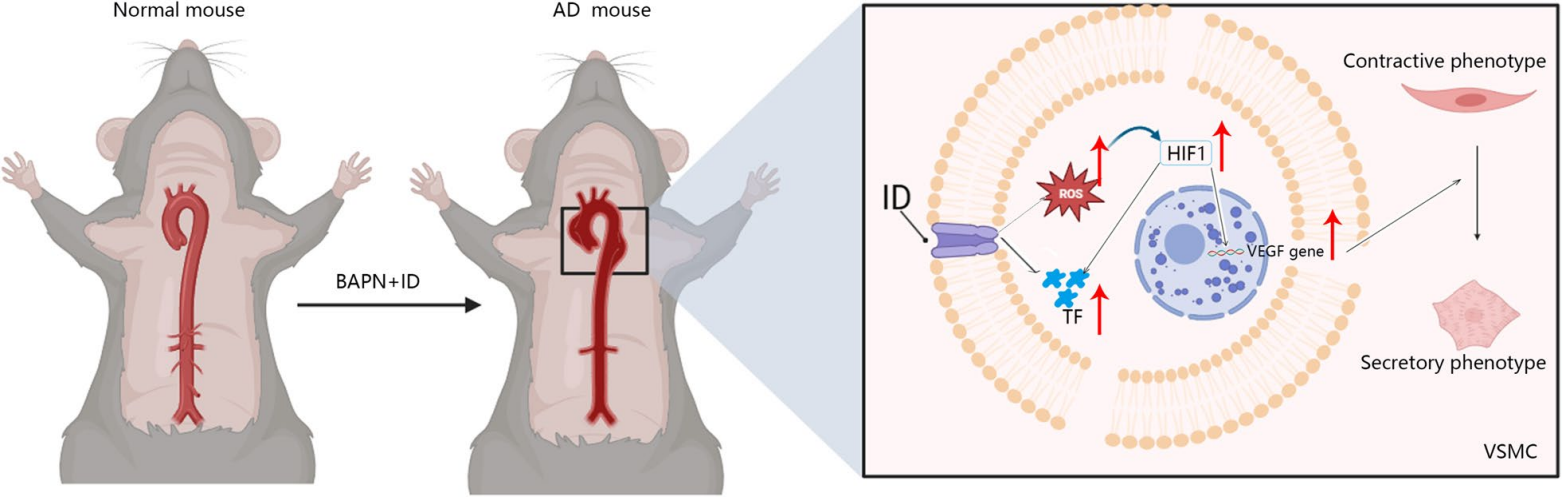
Graphics summarizes that ID promotes ROS expression and activates HIF1 signaling pathway. After activation, HIF1 enters the nucleus and participates in the transcription and expression of VEGF gene, thereby regulating the transformation of VSMC from contractility to synthesis. Phenotypic transformation of VSMC leads to the development of aortic dissection in mice

Aortic dissection, a life-threatening condition, is primarily managed through surgical intervention due to the absence of viable pharmacological treatment options (Tadros et al. 2019; Zhu et al. 2020). The prognosis is unfavorable, and the quality of life remains unsatisfactory; therefore, it is imperative to investigate the underlying pathological changes associated with AD occurrence and conduct fundamental research on potential pharmacological interventions that can ameliorate its progression. This study demonstrates the crucial role of trace element iron in maintaining aortic health within the body (Klip et al. 2017). Iron deficiency triggers hypoxia-induced activation of HIF1, which subsequently regulates downstream gene transcription alterations, promotes phenotypic transformation of VSMCs, and exacerbates AD severity (Liu et al. 2023). By inhibiting HIF1 activity, it is possible to ameliorate VSMC phenotype transformation and thereby decelerate disease progression while extending survival in mouse models. Given its prevalence among humans as a nutritional state, iron deficiency plays a pivotal role in cardiovascular diseases (Janbandhu et al. 2022). Our previous investigation revealed that iron deficiency worsens aortic expansion by disrupting cytoskeletal integrity (Prabhakar et al. 2020). Osteopontin (OPN) can promote inflammation by promoting the chemotaxis and adhesion of macrophages and T cells, and increase the cell-mediated underwear buying process. OPN can also participate in the activation of MMP-9 precursors to promote the transformation of VSMC from systolic to synthetic. Phenotypic switching of VSMCs from contractile cells to secretory and proliferative cells constitutes the primary underlying mechanism for AMD; however, how iron deficiency modulates gene expression and transcription in VSMCs leading to altered phenotypic transformation remains unknown (Yao et al. 2021). In this experiment, we initially explored the impact of iron deficiency on AD occurrence and established an association between iron levels and AD pathogenesis across human samples, animal models, and cell experiments. Results indicate that iron significantly contributes to maintaining the contractile form of human VSMCs based on these investigations. The overall idea of this study is shown in Fig. 6.

The results show that ID can promote the increase of ROS content in cells, while HIF1 plays a role as a transcription factor that is stimulated by hypoxic environment and its expression is increased (Waypa and Schumacker 2019; Cheu et al. 2023). When the aorta is in ID and subjected to external stimulation such as AngII, it will rapidly promote the phenotypic transformation of cells, resulting in increased disease occurrence and expanded lesion involvement. Since iron is an important component of hemoglobin, which is the main component of transporting oxygen, ID and hypoxia should be closely related (Corradi et al. 2023). Our experimental results also prove that the increase of ROS and 8-OHdg in the body during ID induces oxidative stress. HIF is sensitive to hypoxia, so we speculate that ID may affect the expression of HIF by regulating oxygen levels in the body.

So how does HIF1 function as a hypoxic transcription factor in the ID environment? According to transcriptome sequencing results, the expression of HIF1 increases in ID environment and leads to activation of multiple downstream signals (Silagi et al. 2021; March-Diaz et al. 2021). According to the sequencing results, HIF1 also has a certain feedback effect on TF, and also affects the functional structure of smooth muscle cells (Xue et al. 2023). Therefore, HIF1 can not only affect iron metabolism, but also affect the functional structure of cells downstream. According to HIF1 signaling pathway, VEGF is most closely associated with the development of AD (Jiang et al. 2019; Wang et al. 2023).Previous studies have shown that VEGF is closely related to AD and can regulate the phenotypic transformation of smooth muscle cells to promote disease occurrence (Ren et al. 2020).As a transcription factor, HIF1 can enter the nucleus, regulate the transcription expression of VEGF and affect the phenotypic transformation of smooth muscle cells by binding to the DNA in the nucleus (Nazari-Khanamiri and Ghasemnejad-Berenji 2022). When HIF1 is inhibited, the exacerbation of the disease caused by iron deficiency is alleviated (Li et al. 2019; Patko et al. 2023). Therefore, we concluded that ID can affect the phenotypic transformation of VSMC by regulating the HIF1 signal axis, thus affecting the progression of AD. Rational iron supplementation and HIF1 inhibitors may be a new drug therapy to delay AD.

This experiment still has some problems that can be improved. For example, we have proved that ID may regulate oxidative stress to affect the expression of HIF1 (Zhang et al. 2022). Can iron directly participate in the synthesis and function of transcription factors as a cofactor? As a transcription factor, HIF1 can regulate the expression of multiple genes, and we only adopted VEGF as the downstream target protein according to the sequencing results (Xu et al. 2022). Whether HIF1 affects phenotypic transformation of aortic smooth muscle cells through other pathways remains to be further explored (Cao et al. 2022).

## Conclusion

1. Iron deficiency leads to an increase in oxidative stress levels in the aorta, thereby stimulating the expression of HIF1.
2. HIF1 induces the expression of downstream proteins including eNOS/iNOS/VEGF, thereby contributing to alterations in vascular functionality.
3. The up-regulation of VEGF facilitates the phenotypic transition of vascular smooth muscle cells (VSMCs) from a contractile phenotype to a synthetic phenotype, thereby expediting the progression of AD.

## Data Availability

The original data used to support the findings of this study are available from the corresponding author upon request.

## References

[CR1] Akutsu K (2019). Etiology of aortic dissection. Gen Thorac Cardiovasc Surg.

[CR2] Camaschella C (2019). Iron deficiency. Blood.

[CR3] Cao X, Wu W, Wang D (2022). Glycogen synthase kinase GSK3α promotes tumorigenesis by activating HIF1/VEGFA signaling pathway in NSCLC tumor. Cell Commun Signal.

[CR4] Chen Z, Wang C, Yu N (2019). NF2 regulates oxidative stress-induced apoptosis in epidermal HaCaT cells by modulating the HIF1 signaling pathway. Biomed Pharmacother.

[CR5] Cheu JW, Chiu DK, Kwan KK (2023). Hypoxia-inducible factor orchestrates adenosine metabolism to promote liver cancer development. Sci Adv.

[CR6] Corradi F, Masini G, Bucciarelli T (2023). Iron deficiency in myocardial ischaemia: molecular mechanisms and therapeutic perspectives. Cardiovasc Res.

[CR7] Cyran AM, Zhitkovich A (2022). HIF1, HSF1, and NRF2: oxidant-responsive trio raising cellular defenses and engaging immune system. Chem Res Toxicol.

[CR8] Evangelista A, Isselbacher EM, Bossone E (2018). Insights from the international registry of acute aortic dissection: a 20-year experience of collaborative clinical research. Circulation.

[CR9] Janbandhu V, Tallapragada V, Patrick R (2022). Hif-1a suppresses ROS-induced proliferation of cardiac fibroblasts following myocardial infarction. Cell Stem Cell.

[CR10] Jiang Y, Zhou J, Zou D (2019). Overexpression of limb-bud and heart (LBH) promotes angiogenesis in human glioma via VEGFA-mediated ERK signalling under hypoxia. EBioMedicine.

[CR11] Klip IT, Voors AA, Swinkels DW (2017). Serum ferritin and risk for new-onset heart failure and cardiovascular events in the community. Eur J Heart Failure.

[CR12] Knutson AK, Williams AL, Boisvert WA (2021). HIF in the heart: development, metabolism, ischemia, and atherosclerosis. J Clin Invest.

[CR13] Li M, Dai N, Wang D (2019). Distinct APE1 activities affect the regulation of VEGF transcription under hypoxic conditions. Comput Struct Biotechnol J.

[CR14] Li B, Wang Z, Hong J (2021). Iron deficiency promotes aortic medial degeneration via destructing cytoskeleton of vascular smooth muscle cells. Clin Transl Med.

[CR15] Liu L, Hao M, Zhang J (2023). FSHR-mTOR-HIF1 signaling alleviates mouse follicles from AMPK-induced atresia. Cell Rep.

[CR16] March-Diaz R, Lara-Ureña N, Romero-Molina C (2021). Hypoxia compromises the mitochondrial metabolism of Alzheimer’s disease microglia via HIF1. Nat Aging.

[CR17] Nazari-Khanamiri F, Ghasemnejad-Berenji M (2022). Resveratrol may ameliorate rheumatoid arthritis via the STAT3/HIF-1/VEGF molecular pathway. J Food Biochem.

[CR18] Pan L, Bai P, Weng X (2022). Legumain is an endogenous modulator of integrin αvβ3 triggering vascular degeneration, dissection, and rupture. Circulation.

[CR19] Patko E, Szabo E, Vaczy A (2023). Protective effects of pituitary adenylate-cyclase-activating polypeptide on retinal vasculature and molecular responses in a rat model of moderate glaucoma. Int J Mol Sci.

[CR20] Prabhakar NR, Peng YJ, Nanduri J (2020). Hypoxia-inducible factors and obstructive sleep apnea. J Clin Invest.

[CR21] Ren F, Wu K, Yang Y (2020). Dandelion polysaccharide exerts anti-angiogenesis effect on hepatocellular carcinoma by regulating VEGF/HIF-1α expression. Front Pharmacol.

[CR22] Shen YH, LeMaire SA, Webb NR (2020). Aortic aneurysms and dissections series. Arterioscler Thromb Vasc Biol.

[CR23] Shi F, Wang Z (2020). Acute aortic dissection surgery: hybrid debranching versus total arch replacement. J Cardiothorac Vasc Anesth.

[CR24] Silagi ES, Schipani E, Shapiro IM (2021). The role of HIF proteins in maintaining the metabolic health of the intervertebral disc. Nat Rev Rheumatol.

[CR25] Tadros RO, Tang GH, Barnes HJ (2019). Optimal treatment of uncomplicated type B aortic dissection: JACC review topic of the week. J Am Coll Cardiol.

[CR26] Wang L, Li J, Wang Y (2023). Dan-Deng-Tong-Nao softgel capsule promotes angiogenesis of cerebral microvasculature to protect cerebral ischemia reperfusion injury via activating HIF-1α-VEGFA-Notch1 signaling pathway. Phytomedicine.

[CR27] Waypa GB, Schumacker PT (2019). Roles of HIF1 and HIF2 in pulmonary hypertension: it all depends on the context. Eur Respir J.

[CR28] Xiao M, Xian C, Wang Y (2023). Nuciferine attenuates atherosclerosis by regulating the proliferation and migration of VSMCs through the Calm4/MMP12/AKT pathway in ApoE(−/−) mice fed with High-Fat-Diet. Phytomedicine.

[CR29] Xu T, Hu X, Yang G (2022). HIF-1alpha/VEGF pathway mediates 1,3,6,8-tetrabromo-9 H-carbazole-induced angiogenesis: a potential vascular toxicity of an emerging contaminant. J Hazard Mater.

[CR30] Xue Q, Kang R, Klionsky DJ (2023). Copper metabolism in cell death and autophagy. Autophagy.

[CR31] Yao RQ, Ren C, Xia ZF (2021). Organelle-specific autophagy in inflammatory diseases: a potential therapeutic target underlying the quality control of multiple organelles. Autophagy.

[CR32] Zhang L, Li M, Wang Z (2022). Cardiovascular risk after SARS-CoV-2 infection is mediated by IL18/IL18R1/HIF-1 signaling pathway axis. Front Immunol.

[CR33] Zhu Y, Lingala B, Baiocchi M (2020). Type A aortic dissection-experience over 5 decades: JACC historical breakthroughs in perspective. J Am Coll Cardiol.

